# Eye movement characteristics of emotional face recognizing task in patients with mild to moderate depression

**DOI:** 10.3389/fnins.2024.1482849

**Published:** 2024-11-13

**Authors:** Qian Yang, Yanyan Fu, Qiuli Yang, Dongqing Yin, Yanan Zhao, Hao Wang, Han Zhang, Yanran Sun, Xinyi Xie, Jian Du

**Affiliations:** ^1^Institute of Basic Research in Clinical Medicine, China Academy of Chinese Medical Sciences, Beijing, China; ^2^Beijing Anding Hospital, Capital Medical University, Beijing, China; ^3^Institute of Acupuncture and Moxibustion, China Academy of Chinese Medical Sciences, Beijing, China; ^4^Shandong University of Traditional Chinese Medicine, Jinan, Shandong, China; ^5^Jiangxi University of Chinese Medicine, Nanchang, China

**Keywords:** eye movement, depression, AOI, emotional facial expression recognition, cognitive deficit

## Abstract

**Objective:**

Depression is a complex affective disorder characterized by high prevalence and severe impact, commonly presenting with cognitive impairment. The objective diagnosis of depression lacks precise standards. This study investigates eye movement characteristics during emotional face recognition task (EFRT) in depressive patients to provide empirical support for objective diagnosis.

**Methods:**

We recruited 43 patients with depression (Depressive patients, DP) from a psychiatric hospital and 44 healthy participants (Healthy Control, HC) online. All participants completed an EFRT comprising 120 trials. Each trial presented a gray screen for 800 ms followed by a stimulus image for judgment. Emotions were categorized as positive, neutral, or negative. Eye movement trajectories were recorded throughout the task. Latency of First Fixation (LFF), Latency of First Fixation for Eye AOI, and Latency of First Fixation for Mouth AOI were used as representative indicators of early attention, Proportion of Eye AOI, and Proportion of Mouth AOI as measures of intermediate attention, Accuracy (ACC) and Reaction Time (RT) as behavioral indicators of late-stage attention. In this study, these metrics were employed to explore the differences between patients with depression and healthy individuals.

**Results:**

Compared to healthy participants, individuals with depression exhibit longer first fixation latencies on the eyes and mouth during the early attention stage of emotional face recognition, indicating an avoidance tendency toward key facial recognition cues. In the mid-to-late attention stages, depressive individuals show an increased fixation ratio on the eyes and a decreased fixation ratio on the mouth, along with lower accuracy and longer response times. These findings suggest that, relative to healthy individuals, individuals with depression have deficits in facial recognition.

**Conclusion:**

This study identified distinct attention patterns and cognitive deficits in emotional face recognition among individuals with depression compared to healthy individuals, providing an attention-based approach for exploring potential clinical diagnostic markers for depression.

## 1 Introduction

Depression is a complex affective disorder with significant emotional, cognitive, and physical symptoms, characterized by persistent low mood, reduced activity, and slowed cognitive function. According to the Global Burden of Disease Study, approximately 280 million people worldwide suffer from depression, including 5% of adults and 5.7% of individuals over 60, and 25% increase has been triggered by COVID-19 (World Health Organization, 2022). Depression is significantly associated with disability and can lead to severe consequences, including suicide, negatively impacting individuals’ mental and physical health (Iancu et al., 2020; Morin et al., 2020).

Most patients with depression experience cognitive dysfunction, including deficits in executive function, attention, memory, and processing speed (Yan and Li, 2018). These impairments manifest as reduced cognitive flexibility, decision-making, and inhibitory control, along with difficulties in maintaining attention, short-term memory loss, and slower reaction times (Hollon et al., 2006; Koenig et al., 2014; Lee et al., 2012; Wagner et al., 2012). Depression also causes a negative emotional bias, where individuals exhibit a preference for negative stimuli, leading to the misinterpretation of information through a negative lens. This bias is linked to deeply ingrained negative self-schemas that sustain depressive symptoms (Jiang, 2024). Studies show depressed individuals have slower reaction times when recognizing facial expressions, particularly neutral ones, and reduced accuracy in identifying positive expressions, often mistaking them for neutral or negative (Leppanen et al., 2004; Sfarlea et al., 2018). However, they generally retain the ability to recognize sad expressions (Dalili et al., 2015).

The clinical diagnosis of depression primarily relies on patients’ clinical symptoms supplemented by depression-related scale scores, lacking specific physiological and biochemical indicators as auxiliary diagnostic criteria (Cuijpers et al., 2020). This absence of a “gold standard” akin to the diagnosis of other organic diseases has prompted numerous studies in recent years to explore objective diagnostic indicators for depression using physiological signals, biochemical markers, and facial visual features (Byun et al., 2019; Koller-Schlaud et al., 2020; Rushia et al., 2020; Thoduparambil et al., 2020; Xing et al., 2019; Du et al., 2022).

The diagnostic approach based on facial visual features objectively assesses the severity of depression by analyzing relevant information from the patient’s face. It further summarizes behavioral characteristics specific to individuals with depression to guide clinical diagnoses made by doctors. The equipment required for this method is simple—a camera—making it cost-effective and easily accessible. Importantly, subjects do not need direct contact with the equipment during data collection, allowing them to maintain a natural state of mind without any hindrance and ensuring genuine mental state data can be captured. Some scholars believe that this method is particularly beneficial for patients experiencing reduced interest or pleasure due to its user-friendly nature. Consequently, it holds significant research value and potential for development (Du et al., 2022).

Recently, there have been significant findings from eye movement experiments conducted on patients with depression. In free-viewing tasks, patients with depression show fewer fixation points and shorter total fixation times on positive images compared to healthy controls. Additionally, the transition time from negative to neutral stimuli was longer in the depression group than in the healthy group (Qian et al., 2019). In dot-probe tasks, it was observed that after undergoing positive word training, patients with depression showed a significant reduction in both the number and duration of fixations on the negative portion of pictures when compared to their pre-training performance (Liu et al., 2015). In recent years, an increasing number of researchers have integrated machine learning algorithms into modeling various indicators derived from eye movement experiments as well as other cross-modal indicators. The aim is to identify a combination method that yields optimal recognition rates for diagnosing depression (Pan et al., 2019; Shen et al., 2021; Wang et al., 2018).

Therefore, this study focuses specifically on individuals with mild to moderate depression and utilizes eye tracking technology to investigate different eye movement indicators during an emotional face recognition task among depressive patients. A comparison will be made against healthy individuals to enhance support for behavioral experimental data related to objective diagnostic indicators for depression while providing valuable reference data for future research involving emotional face recognition and eye movements among depressive patients.

## 2 Materials and methods

### 2.1 Participants

All participants were required to have normal or corrected normal vision, as well as be free from color blindness or color weakness to eliminate any potential impact of visual impairments on the experiment. Participants were aged between 18 and 60, right-handed, and were required to read the experimental instructions, agree to the procedures, and sign an informed consent form.

Depressed patients were recruited from a tertiary psychiatric hospital in Beijing. They were assessed by 2–3 psychiatrists, including one senior psychiatrist, using the 17-item Hamilton Depression Rating Scale (HAMD-17), and clinically diagnosed according to the criteria for depression outlined in the *Diagnostic and Statistical Manual of Mental Disorders, Fifth Edition* (DSM-5). Patients scoring >7 on the HAMD-17 and clinically diagnosed with depression were included in the depression group, while individuals with other psychiatric disorders such as schizophrenia, anxiety disorders, or bipolar disorder were excluded.

The healthy control group was recruited online via social media platforms and consisted of volunteers. In addition to meeting the vision, age, and handedness requirements, healthy participants were required to score < 7 on the HAMD-17 and have a total score of <160 on the Symptom Checklist-90 (SCL-90).

A total of 45 participants were recruited for the depression group and 51 for the healthy control group. Due to data quality issues, 2 depressed participants and 7 healthy participants were excluded, resulting in a final sample of 87 participants, with 43 in the depression group and 44 in the healthy control group.

This study was approved by the Ethics Committee of the Institute of Clinical Basic Medicine, China Academy of Chinese Medical Sciences, under the approval number P23009/PJ09.

### 2.2 Experimental design and data collection

#### 2.2.1 Emotional images

The emotional facial images used in the Emotional Face Recognition Task were sourced from the Chinese Affective Face Picture System (CAFPS), developed by Professor Yuejia Luo’s team at Shenzhen University (Xu et al., 2011). All images were standardized in terms of color, brightness, and size and demonstrated good reliability and validity. A total of 120 images were selected from the CAFPS database for the experiment, with 40 images for each emotional category (positive, neutral, negative). The images were matched for gender, with 20 male and 20 female faces in each emotional category.

#### 2.2.2 Experimental instruments and software

**Figure 1:**
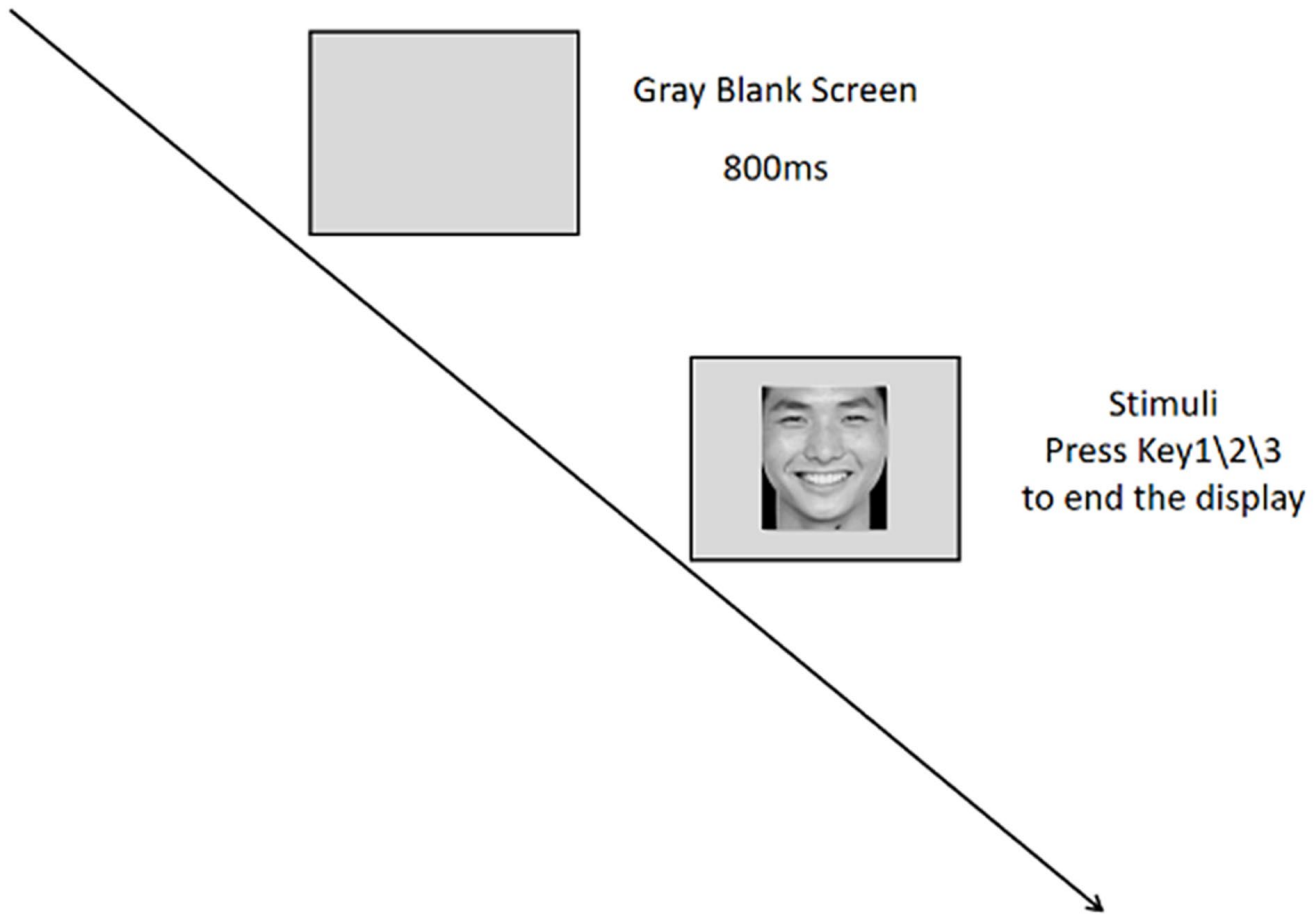
Processing diagram of the single trial.

The experiment employed a Tobii Pro X3-120 eye tracker produced by Tobii Technology AB, Sweden. The device is 324 mm in length, weighs 118 g, and operates at a sampling rate of 120 Hz. It can be directly mounted on monitors or laptops with screens up to 25 inches using the accompanying adhesive stand (as shown in Figure 1). During the experiment, participants were seated approximately 65 cm from the screen. Fixations were calculated using Tobii’s built-in I-VT algorithm, which automatically identified fixations with a duration greater than 60 ms. Adjacent fixations separated by less than 75 ms and with an angular distance of less than 0.5 degrees were merged into a single fixation point. Eye-tracking data were collected and exported using Tobii Pro Lab Version 1.152. Behavioral data were collected, merged, and exported using E-Prime 3.0. Data preprocessing and statistical analysis were conducted using R Studio version 4.3.2, and data visualization was performed using GraphPad Prism version 10.1.2.

#### 2.2.3 Experimental design and procedure

The experiment followed a 2 (group: depression patients/healthy controls) × 3 (emotional face type: positive/neutral/negative) mixed factorial design. Participants were required to complete the Emotional Face Recognition Task (EFRT), in which they were asked to identify the emotional attributes of randomly presented facial images. Meanwhile, the eye tracker recorded their eye movement trajectories.

Before the experiment, the eye-tracking equipment was calibrated. Upon arrival at the laboratory, participants were briefed on the task and informed of the relevant precautions. They then performed the Emotional Face Recognition Task. The experiment took place in a soundproof, windowless room, with the main light source being a ceiling light in the center. The computer faced the wall with its back to the light source to ensure soft, non-reflective lighting conditions.

The eye-tracking experiment followed an event-related design with a total of 120 trials presented in random order. The flow of a single trial is illustrated in Figure 1. Each trial began with a blank gray screen displayed for 800 ms, followed by the random presentation of an emotional face image at the center of the monitor. The duration of each image presentation was not fixed and depended on the participant’s response. Participants were required to make a judgment by pressing one of three keys: “1” for positive, “2” for neutral, and “3” for negative. Once a selection was made, the trial ended, and the next trial began.

### 2.3 Data preprocessing and eye movement index calculations

#### 2.3.1 Zoning and labeling of AOI

**Figure 2:**
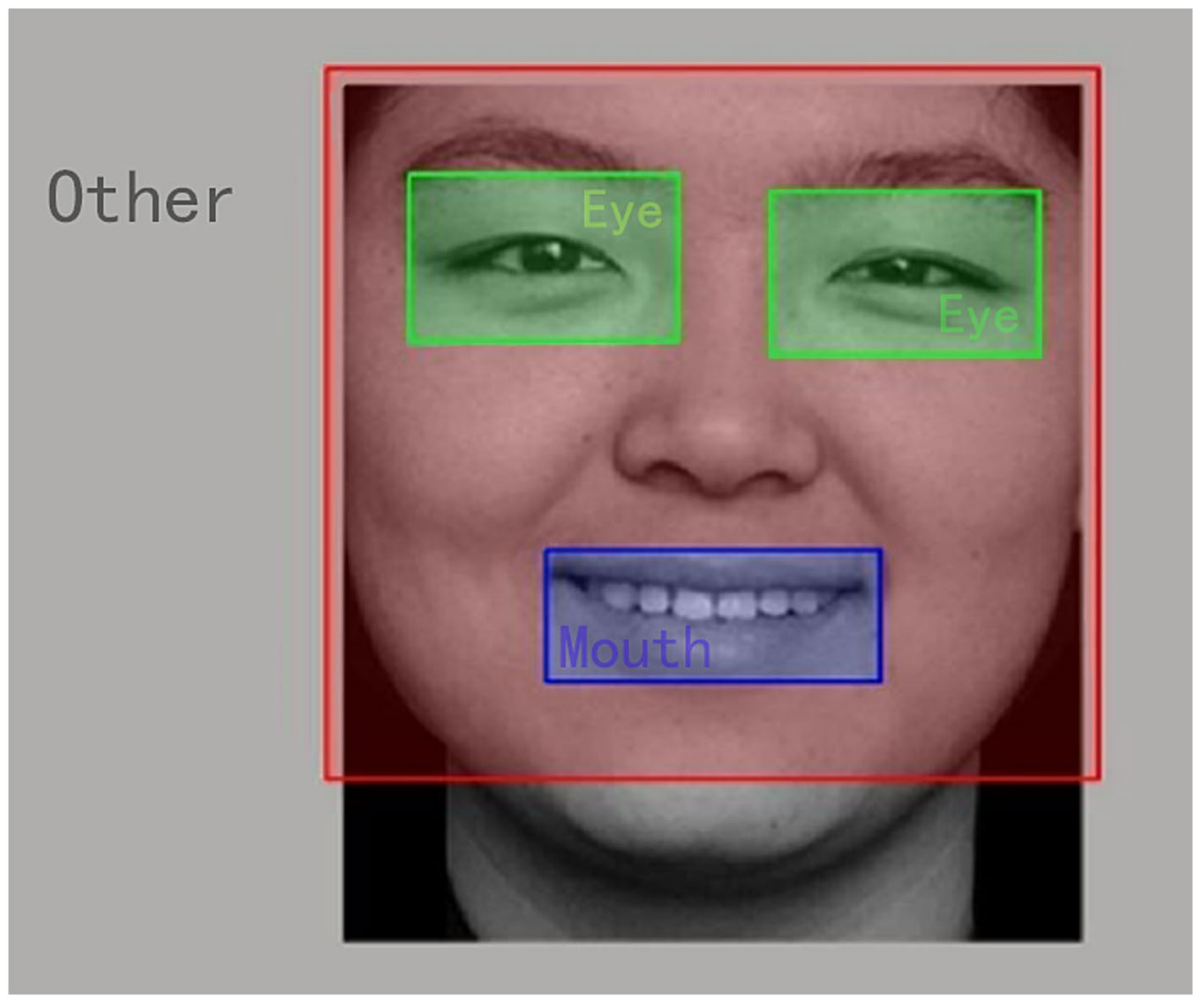
The effect diagram of AOI division.

In Python, the shape key point coordinate prediction tool of the DLIB library and the standardized 68-point facial feature point calibration model are used to automatically identify the facial features coordinates of the stimulus images, the location is recorded by drawing and generating AOI coordinates table, and the AOI is judged by comparing the fixation point x and Y coordinates with the AOI coordinates, blue is Mouth, red is Face, and the Other areas are all Other (Figure 2).

#### 2.3.2 Data screening and imputation

In this study, to clearly differentiate between eye-tracking data and reaction time/accuracy data, we referred to the latter as “behavioral data.” Eye-tracking data were first stored in Tobii Pro Lab software, and during export, only data with a valid sampling rate of ≥60% were selected. Invalid sampling points during the experiment could result from undetected eye movements or unclassified samples, such as when participants blinked, closed their eyes, or looked away from the screen. During this process, data from two participants in the depression group were excluded.

Both eye-tracking and behavioral data were preprocessed and analyzed using R. Outliers beyond 1.5 times the interquartile range (IQR) were excluded. Missing values were imputed using mean substitution. After data cleaning, missing values were imputed as follows: Latency of first fixation (12%), LFF of eye (3%), LFF of mouth (3%), Look proportion of eye (2%), Look proportion of mouth (0%), Accuracy (9%), and Reaction time (5%).

### 2.4 Data analysis

In this study, the analyzed data primarily comprised eye-tracking and behavioral data. Eye-tracking metrics were derived from the raw data, including the latency of the first fixation (LFF) for each trial, the LFF of the eye AOI (area of interest), the LFF of the mouth AOI, the proportion of fixation time on the eye AOI, and the proportion of fixation time on the mouth AOI. Behavioral data included reaction time and accuracy.

#### 2.4.1 Eye-tracking metrics calculation

First, the timestamps from the raw data were used to record the stimulus start time, end time, and the start and end times of each fixation point. Statistical metrics were then calculated using the following formulas.

##### 2.4.1.1 Latency of first fixation

LFF-related eye-tracking metrics reflect early visual attention characteristics. By analyzing the LFF in different AOIs, early attention preferences can be inferred.

###### 2.4.1.1.1 LFF for each trial

Calculated as the time from the stimulus onset (STS, Start Time of Stimulus) to the first fixation onset (STFF, Start Time of First Fixation). This measures the time from when the image appears until the first meaningful fixation occurs. Note that fixations that started before but ended after stimulus onset were excluded to ensure that the first fixation was drawn by the stimulus image.

###### 2.4.1.1.2 LFF for eye AOI

Time from the stimulus onset to the first fixation on the eye AOI.

###### 2.4.1.1.3 LFF for mouth AOI

Time from the stimulus onset to the first fixation on the mouth AOI.

##### 2.4.1.2 Proportion of look time

Fixation proportion is calculated by measuring the fixation time in different AOIs. Fixation duration is a common metric for mid-term fixation analysis, but since the stimulus presentation time was not fixed in this study, fixation duration is highly correlated with reaction time, making it less suitable as a statistical metric. Instead, we converted fixation duration to a proportion, allowing a comparison of depression characteristics across different AOIs.

###### 2.4.1.2.1 Look proportion of eye

First, the fixation duration for each AOI in a single trial was calculated, then summed to get the total fixation duration (total look time). The fixation proportion for each AOI was then calculated by dividing the fixation duration for that AOI by the total fixation duration. Look proportion of eye = Look time of eye AOI/Total look time

###### 2.4.1.2.2 Look proportion of mouth

Similar to the above, Look proportion of mouth = Look time of mouth AOI/Total look time.

#### 2.4.2 Statistical analysis

For demographic data, age differences between the two groups were analyzed using a *t*-test, and gender differences were compared using a chi-square test. Since age differences were significant, age was controlled as a covariate in the analysis of eye-tracking and behavioral data, using a 2×3 ANCOVA design. *Post-hoc* tests and simple effects analyses were corrected using the Bonferroni method for *p*-values.

## 3 Results

### 3.1 Demographic information

Demographic information was statistically presented in Table 1, there was no difference in gender composition between the DP group and the HC group, however, the age of the DP group was significantly larger than that of the HC group, so the covariance analysis was used to count the dependent variable and the age was a covariate to control for the subsequent statistics.

**Table 1:** Demographic information for DP and HC.

	Group		χ^2^/T	*p*-value
	DP (*n* = 43)	HC (*n* = 44)		
Gender (M/F)	(6/20)	(3/14)	χ^2^ = 0.851	0.356
Age	39.6 ± 13.6	26.8 ± 2.62	T = 6.095	<0.001***

### 3.2 Analysis of covariance for statistical indicators

Table 2 showed the descriptive statistical table of the values of each dependent variable in the DP group and the HC group under different emotional face conditions, and the summary table of the results of analysis of covariance for all dependent variables was shown in Table 3.

**Table 2:** Descriptive statistical analysis.

Types of facial emotions	Positive		Neutral		Negative	
Group	DP(*N* = 43)	HC(*N* = 44)	DP(*N* = 43)	HC(*N* = 44)	DP(*N* = 43)	HC(*N* = 44)
Latency of first fixation (s)	0.26 ± 0.08	0.24 ± 0.06	0.27 ± 0.08	0.25 ± 0.07	0.25 ± 0.07	0.24 ± 0.07
Latency of first fixation for eye AOI (s)	1.04 ± 0.43	0.81 ± 0.42	0.82 ± 0.37	0.71 ± 0.32	0.92 ± 0.42	0.71 ± 0.35
Latency of first fixation for mouth AOI (s)	1.26 ± 0.57	1.05 ± 0.46	1.73 ± 0.66	1.33 ± 0.55	1.48 ± 0.61	1.20 ± 0.56
Look proportion of eye AOI	0.31 ± 0.11	0.31 ± 0.11	0.38 ± 0.13	0.34 ± 0.12	0.34 ± 0.11	0.33 ± 0.11
Look proportion of mouth AOI	0.22 ± 0.07	0.24 ± 0.07	0.17 ± 0.06	0.20 ± 0.07	0.18 ± 0.07	0.22 ± 0.06
Accuracy	0.96 ± 0.06	0.97 ± 0.04	0.89 ± 0.07	0.93 ± 0.07	0.91 ± 0.08	0.96 ± 0.04
Reaction time(s)	1.30 ± 0.45	1.03 ± 0.31	1.49 ± 0.43	1.21 ± 0.36	1.44 ± 0.43	1.07 ± 0.27

**Table 3:** Covariance analysis table.

	Main effect of group			Main effect of type of emotional images			Main effect of age			Interaction effect of group and type of emotional images		
	*F*(1,85)	*P*	η^2^p	*F*(2,85)	*P*	η^2^p	*F*(1,85)	*P*	η^2^p	*F*(2,85)	*P*	η^2^p
Latency of first fixation	1.599	0.207	0.012	0.112	0.894	0.001	1.675	0.197	0.007	0.444	0.642	0.003
Latency of first fixation for eye AOI	17.519	0.000	0.016	2.411	0.092	0.019	6.920	0.009	0.027	0.105	0.901	0.001
Latency of first fixation for mouth AOI	21.329	0.000	0.027	11.261	0.000	0.081	4.687	0.031	0.018	1.607	0.203	0.012
Look proportion of eye AOI	4.819	0.029	0.048	4.029	0.019	0.031	10.075	0.002	0.038	1.373	0.255	0.011
Look proportion of mouth AOI	11.791	0.001	0.033	8.822	0.000	0.065	1.034	0.869	0.000	1.034	0.357	0.008
Accuracy	27.654	0.000	0.036	5.286	0.000	0.062	0.745	0.022	0.020	0.745	0.476	0.006
Reaction time	49.430	0.000	0.060	10.539	0.001	0.055	1.136	0.001	0.040	1.136	0.323	0.009

The main effect of GROUP was not significant in the 2 × 3 ANCOVA with the LFF as the dependent variable, but it was significant in the ANCOVA with the LFF of Eye AOI [*F*(1, 85) = 17.519, *p* < 0.001, η^2^p = 0.016] and LFF of Mouth AOI [*F*(1, 85) = 21.329, *p* < 0.001, η^2^p = 0.027]. The first fixation latency of Eye AOI and Mouth AOI in DP group was significantly longer than HC group.

In the ANCOVA analysis of look proportion, the main effects of GROUP were significant both in Eye AOI [*F*(1, 85) =4.819, *p* = 0.029, η^2^p = 0.048] and Mouth AOI [*F*(1, 85) =11.791, *p* = 0.001, η^2^p = 0.033], the post-hoc test showed that the proportion of Mouth AOI in DP group is significantly lower than HC group.

The main effect of GROUP was also significant in the analysis of Accuracy [*F*(1, 85) =27.654, *p* < 0.001, η^2^p = 0.036] and Reaction Time [*F*(1, 85) =49.430, *p* < 0.001, η^2^p = 0.060], the *post-hoc* test showed that the accuracy of EFRT in DP group was significantly lower than HC group, and the reaction time in DP group was significantly longer than HC group.

## 4 Discussion

### 4.1 Avoidance of recognition cues in early attention

**Figure 3:**
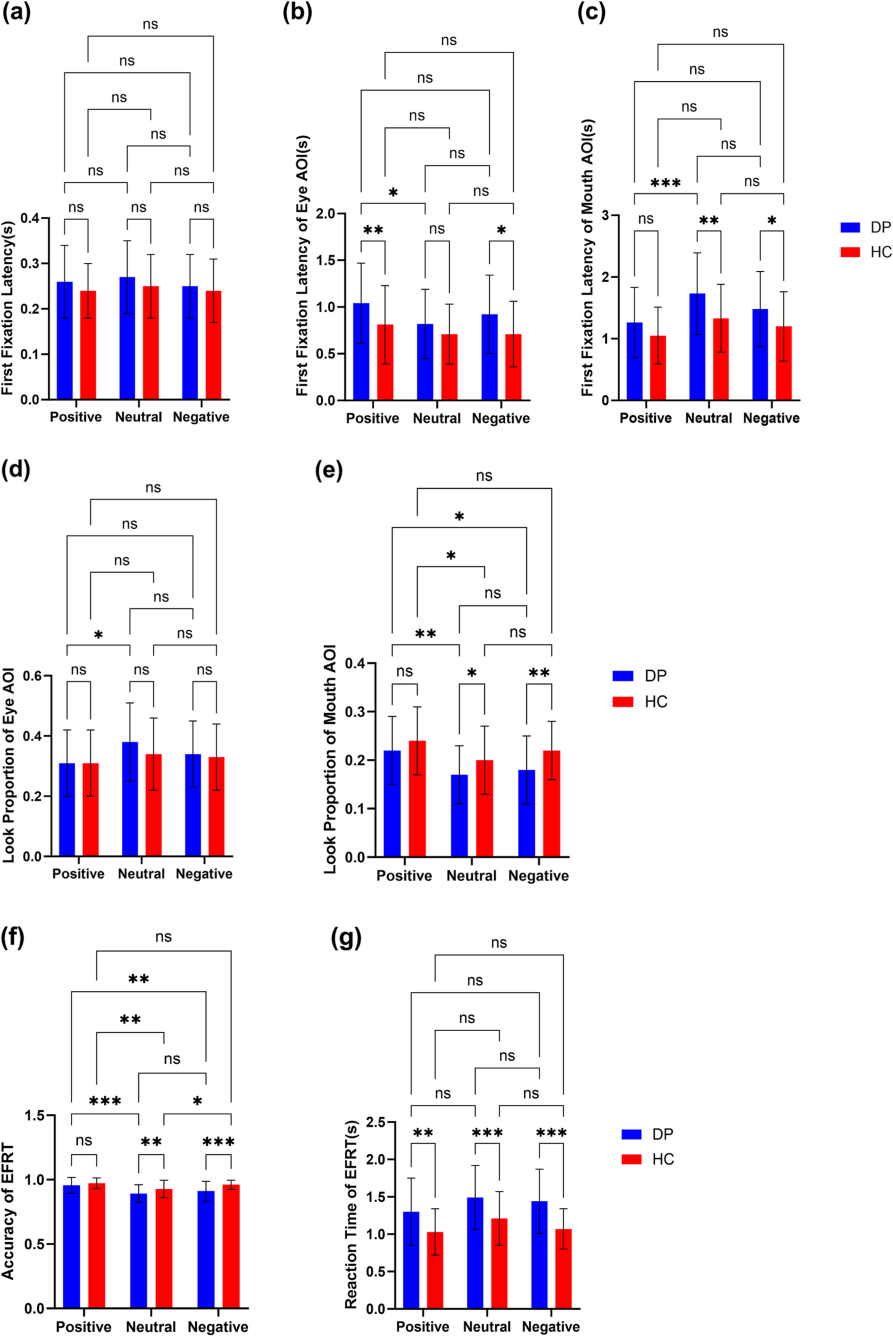
(a–c) Latency of first fixation in EFRT(s). (d,e) Look proportion of AOIs (eye and mouth) in EFRT. (f,g) Accuracy and reaction time(s) of EFRT. ***p < 0.001; **p < 0.01; *p < 0.05; ns, non-significant.

We found some interesting results on the latency of first fixation. For the whole picture (Figure 3a), there was no difference between the DP group and the HC group, but there were some differences when the latency of the first fixation in the different regions of interest was analyzed, people with depression pay attention to the pictures at the same time as healthy people, but they pay attention to the features later than healthy people.

**Figure 4:**
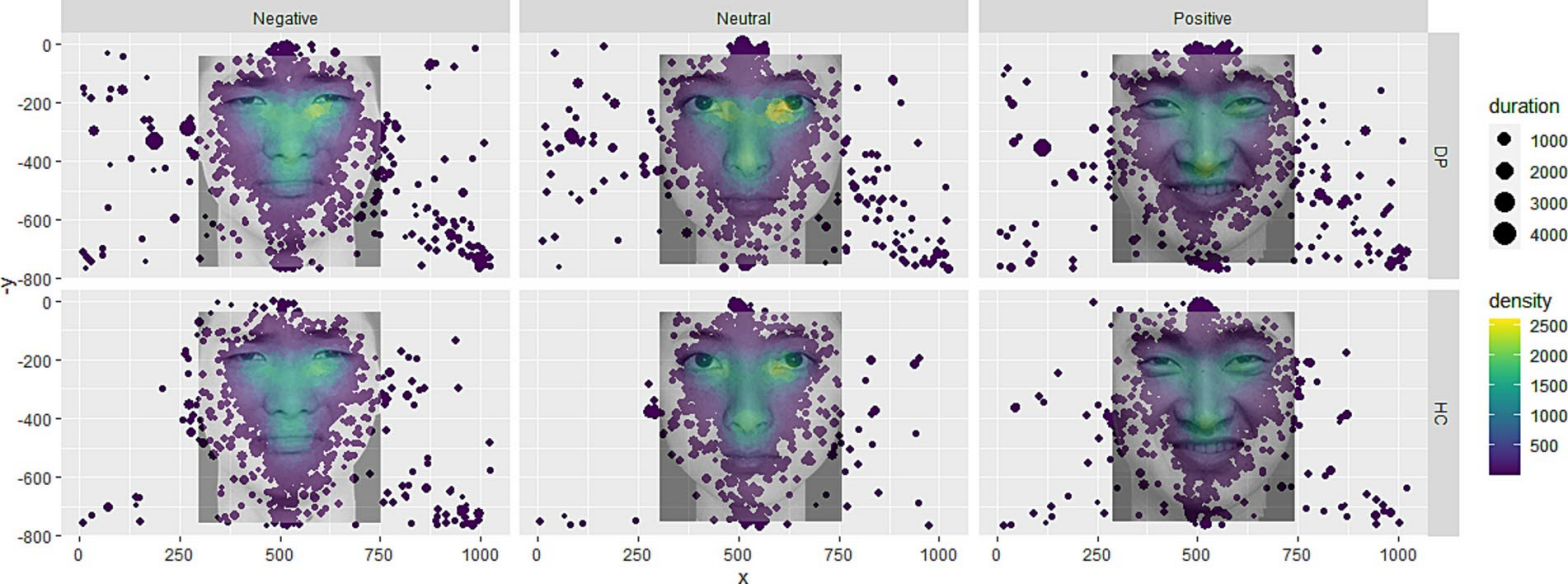
Fixation point heat map. Larger size of the fixation point represents longer fixation time, and the higher the color heat, the higher the density.

**Figure 5:**
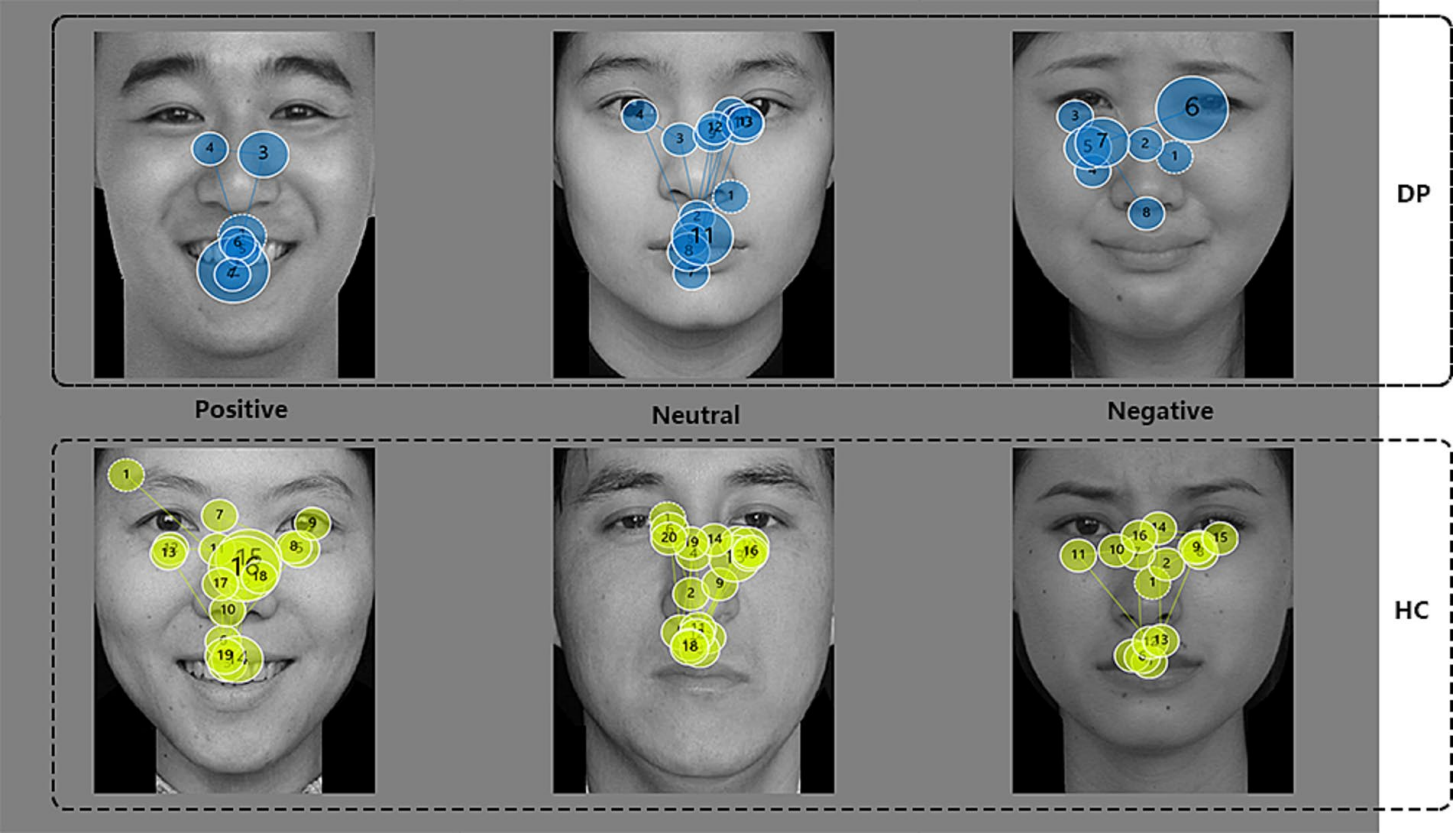
Eye trace images of DP and HC groups. The number in circle represents the order of fixation points, the size of the circle represents the duration of fixation, and the larger the circle, the longer the duration.

Previous studies have shown that most of the implementation of emotional face recognition is focused on the eyes and mouth (Eisenbarth and Alpers, 2011). The eye is a major cue for recognizing negative emotions (Franca et al., 2023; Grainger and Henry, 2020), the latency of the first fixation of the eye was greater in the DP Group than in the HC Group, suggesting that depressed patients are slower to notice the eye site when recognizing expressions, as can be seen from Figure 3b, especially when judging positive and negative faces, the latency of the first fixation point of “Eyes” was significantly greater than that of the healthy group, this suggests that people with depression have significantly more avoidance of the eye region when it comes to recognizing faces with distinct facial expressions than healthy people. This is consistent with findings from a study on the effects of depressive tendencies on eye gaze in social interactions. The study found that while depressive tendencies do not affect attention to others’ faces as a whole, they do influence attention to others’ eyes, suggesting an avoidance of the eye region among individuals with depressive tendencies (Suslow et al., 2024). Furthermore, a comparison within the depression group revealed that the first fixation latency on the eye region was significantly longer for positive emotional faces than for neutral images. This suggests a stronger avoidance of the eye region in response to positive emotional faces among individuals with depression. It may also be due to the role of the eyes as a primary cue for recognizing negative emotions, which could counterbalance avoidance tendencies in response to negative faces, thus making avoidance less prominent for positive faces. Similarly, the first fixation latency on the mouth was longer in the depression group than in the healthy control group, indicating a slower attention shift to the mouth region when identifying emotional faces compared to healthy individuals. As shown in Figure 3c, this delay is particularly evident in responses to neutral and negative expressions, which may be attributed to the mouth’s role as a key indicator of positive emotional expressions (Grainger and Henry, 2020). When judging positive emotional faces, people typically fixate on the mouth first, which might explain why differences in first fixation latency on the mouth between the depression and control groups are less pronounced for positive faces but more noticeable in neutral and negative conditions (Figures 4, 5).

The relevant LFF indicators reveal that, in the early attention stage, individuals with depression do not show a significant difference in response to stimulus images compared to healthy controls. However, they exhibit a certain delay in focusing on effective facial expression cues. This phenomenon reflects, to some extent, a slowing in attention direction and a reduction in efficiency for emotion recognition tasks among individuals with depression.

### 4.2 Altered processing patterns in mid-to-late attention stages and facial recognition deficits

The look proportion is derived from fixation duration, which reflects the difficulty participants experience in completing the task—the longer the duration, the greater the difficulty (Goller et al., 2019). Some discrepancies exist between prior studies and the findings of this study. Most studies suggest that individuals experiencing sadness tend to focus less on the eyes during facial recognition (Hills and Lewis, 2011), and individuals with depression engage in less eye contact during conversations (Fiquer et al., 2018). In social interactions, eye contact represents crucial information in the dialogue, conveying social interest and closeness (Cui et al., 2019). By diverting their gaze from others’ eyes, individuals with depression may avoid deeper social engagement (Hames et al., 2013). The observed differences may be attributed to the specificity of this experimental task. Studies that use look proportion as a measure typically employ a free-viewing paradigm, where participants have ample time to view images. However, in this study, participants were required to respond to stimulus images as quickly as possible, with the images disappearing immediately after the response. Since facial expression recognition generally begins with the eyes and then moves to the mouth (Xue and Yantao, 2007), it is possible that limited viewing time in this study resulted in a focus advantage for the eyes as the first facial feature noticed. Given that both the depressed and healthy groups were under the same task conditions, a more plausible explanation may be an altered facial recognition pattern among individuals with depression.

In this study, Figures 3d,e reveal that individuals with depression show a higher look proportion on the eyes but a significantly lower look proportion on the mouth compared to the healthy control group. This suggests that during the task, participants in the depression group attempt to gather cues more from the eyes and less from the mouth. Research on facial expression recognition patterns in healthy individuals indicates a general preference to seek emotional cues from the mouth, with the eyes providing comparatively less prominent cues (Wang et al., 2011). Thus, these findings may suggest that individuals with depression have an altered facial expression recognition pattern compared to healthy individuals. When considering response time and accuracy, the accuracy rate in the depression group was lower than that in the healthy control group, while response times were longer. This indicates that individuals with depression are not only slower in recognizing emotional faces but also less accurate. These results imply that a facial emotion recognition pattern focused more heavily on the eyes is less effective for individuals with depression than for healthy individuals, highlighting deficits in emotional face recognition among those with depression compared to healthy controls.

## 5 Conclusion

Individuals with depression display certain disadvantages and deficits in attention characteristics during emotional face recognition tasks compared to healthy individuals. Specifically, depressive participants exhibit increased first fixation latency to key facial cue areas (eyes and mouth), indicating an avoidance of effective cues in facial recognition. They also show a decreased look proportion on the mouth and an increased look proportion on the eyes, accompanied by reduced accuracy and prolonged response times. These findings suggest changes in cognitive patterns and deficits in facial recognition during emotional processing in individuals with depression. The results of this study support the hypothesis that individuals with depression have cognitive impairments and facial recognition deficits, offering an attention-based approach to exploring clinical diagnostic markers for depression.

## 6 Limitations and future directions

In recruiting healthy controls and screening them using scales, the healthy participants’ ages were relatively concentrated, creating a disparity with the experimental group. Future research should increase the sample size to match the ages of the healthy control group more closely with the experimental group. What is more important is that the conclusions of this study need further validation in future research.

## Data Availability

The raw data supporting the conclusions of this article will be made available by the authors, without undue reservation.
